# Marginalized Identities and Intersectionalities that Contribute to College Students’ Mental Health Challenges and Use of Psychological Services

**DOI:** 10.1177/10870547251358674

**Published:** 2025-08-11

**Authors:** Sohyun An Kim, Laura V. Rhinehart

**Affiliations:** 1 School of Education and Information Studies, University of California Los Angeles, Los Angeles, CA, USA

**Keywords:** psychological disorders, postsecondary students, marginalized identities, ADHD, first-generation college students, intersectionality

## Abstract

**Objective:**

The rates of college students experiencing mental health challenges are increasing. Students with marginalized identities such as women, first generation college students (FGCS), or students with disabilities may be more at risk, and having multiple marginalized identities can impose added challenges. This study examined students’ identities that contribute to college students with psychological disorders’: (1) severity of mental health challenges, and (2) use of on-campus psychological services.

**Methods:**

A nationwide survey was used to draw a sample of college students with psychological disorders (*N* = 5,120).

**Results:**

For students with psychological disorders, being a woman was associated with heightened mental health challenges, even when other identities were accounted for (i.e., FGCS status, disability status, dual identities). Having ADHD was also associated with heightened mental health challenges when dual identities were not accounted for. Additionally, when students with ADHD’s dual identity as a FGCS was accounted for, students with psychological disorders and ADHD who are also FGCS were found to experience heightened psychological challenges. Moreover, FGCS were less likely to utilize mental health services on campus, even when other identities were accounted for.

**Conclusion:**

Findings highlight the role marginalized identities play for college students with psychological disorders and the importance of considering students’ intersectionality when it comes to mental health and seeking professional support.

College enrollment rates for young adults in the United States are steadily increasing, from 35% in 2000 to 39% in 2018 (Irwin et al., 2024), and these new college classes are comprised of many students with marginalized identities, including students with disabilities, women, and first-generation college students (Baker et al., 2018). As with the enrollment rate, the rate of college students experiencing mental health challenges is also on the rise (Hernández-Torrano et al., 2020; Lipson et al., 2019; Oswalt et al., 2020; Pedrelli et al., 2015). Mental health challenges in college students can impede their academic success and increase their likelihood of dropping out (Auerbach et al., 2016; Duffy et al., 2020; Ishii et al., 2018). Such conditions are also associated with alcohol misuse (Dawson et al., 2005; Levin et al., 2012), social withdrawal (Li et al., 2017), self-injurious behaviors (Gollust et al., 2008; Taliaferro & Muehlenkamp, 2015), and suicidal behaviors (Bakken, 2021; Cramer et al., 2017) in college students.

Not only is the number of students experiencing mental health challenges increasing, but the severity and complexity of these conditions are also on the rise (Hernández-Torrano et al., 2020). Recent data show that approximately one in five college students has a psychological disorder (Auerbach et al., 2016), yet most of these students do not receive the recommended treatment (Blanco et al., 2008; Eisenberg et al., 2011; Rosenthal & Wilson, 2008). There is a strong evidence base that supports the effectiveness on-campus mental health services (see Barnett et al., 2021 for a review). However, when conditions are left untreated, symptoms can intensify, leading to more severe forms of psychological disorders (Ghio et al., 2015).

There are a number of reasons why college students do not seek or receive mental health treatments. Some students believe there is stigma around receiving mental health services (Vidourek et al., 2014). Other students describe a fear of therapy sessions (Linden & Jurdi-Hage, 2017). Finally, some students report wanting to handle the issues alone (Ebert et al., 2019; Janota et al., 2022). While attitudes and beliefs prevent some students from utilizing campus mental health services, other factors such as students’ marginalized identities, impact students’ utilization of services, and these factors are less explored in the literature.

## College Students with Socially Marginalized Identities

College students with marginalized identities are more prone to experiencing psychological disorders (Shepherd et al., 2023). Additionally, the need-to-treatment gap in mental health care is more pronounced in socially marginalized communities, including communities comprised of first-generation college students, lower-income individuals, and/or ethnic/racial minorities (Kook et al., 2025). These marginalized groups are more vulnerable to psychological disorders and may face socially- and culturally-related barriers to accessing campus resources. In addition to aforementioned factors, being a neurodivergent student (i.e., having autism or ADHD), as well as being a woman may also impact students’ mental health challenges and their utilization of psychological services. Importantly, intersectionality of multiple marginalized identities (e.g., being a woman and having ADHD) has been found to have a stronger association with psychological symptoms, compared to systemic or contextual factors alone (Seng et al., 2012).

### Neurodiversity: Autism and ADHD

The prevalence of mental health challenges in the autistic population is substantially higher than in their non-autistic counterparts (Lai et al., 2019). Autistic college students are more likely to experience depression or anxiety, and they are more likely to report self-harm and suicidal behaviors when compared to their non-autistic peers (Campbell et al., 2022; Davis et al., 2021; Fernandes et al., 2021; Jackson et al., 2018; Kuder et al., 2021). There is emerging evidence showing the benefits of psychological services on campus for autistic students (Kuder et al., 2021), yet these students are less likely to access therapeutic services than individuals without autism (Mandy, 2022). A recent systemic review (Adams & Young, 2021) on self-reported barriers to accessing mental health services in individuals with autism identified several important factors. The most prominent barrier was the lack of mental health service providers’ autism-specific knowledge. For example, many participants reported that service providers lacked expertise in treating mental health in autistic individuals (Anderson et al., 2018), and they wished that there were more autism-specific accommodations and flexibility in the delivery methods to meet their autism-related needs (Camm-Crosbie et al., 2019). Although there is a pressing need to offer autism-specific mental health services to autistic college students, another recent study (Nachman et al., 2022) found that autism-specific support programs in postsecondary institutes are few and far between. More importantly, most of these programs are offered at 4-year universities (85.1%) when more autistic students attend 2-year community colleges than 4-year colleges (Newman et al., 2011).

Similarly, individuals with ADHD are at increased risk for psychological disorders (Anastopoulos et al., 2018; Mak et al., 2022; Miyasaka & Nomura, 2022). More specifically, in a nationally representative sample, among adults aged 18 to 44 with ADHD, 38% were identified with a mood disorder, roughly half met criteria for an anxiety disorder, and 15% had a substance abuse disorder (Kessler et al., 2006). Further, college students with ADHD report high rates of depression (Mochrie et al., 2020), tend to binge drink and misuse drugs more frequently than students without ADHD (Mochrie et al., 2020), and they are at a much higher risk of being diagnosed with anorexia or bulimia (Schiros & Antshel, 2023).

Despite these risks, many college students with ADHD are highly successful. There are several factors that contribute to success in college for students with ADHD. One factor is seeking out academic support (DuPaul et al., 2021). Another factor is participating in targeted supports for college students with ADHD. For instance, students who participated in an intervention for college students with ADHD, which included group cognitive behavior therapy and individual mentoring, showed improved emotional and academic outcomes (Anastopoulos et al., 2021; Anastopoulos & King, 2015). Taken together, these findings suggest that various on-campus support services tailored to neurodiverse students can improve outcomes for college students with ADHD and/or autism.

Data show that autism and ADHD often co-occur, with up to 70% of autistic individuals also having ADHD (Rong et al., 2021). Therefore, exploring the impacts of autism and ADHD as well as the impacts of this co-occurrence on students’ mental health challenges and utilization of services may shed light on understanding the unique challenges of each group. The present study explores these factors.

### Female Students

Female students tend to experience more intense levels of mental health challenges when compared to their male counterparts (Auerbach et al., 2019; Eisenberg et al., 2013; Nogueira et al., 2022; Pedrelli et al., 2016). This gender-based disparity in mental health may be related to the fact that women are more likely to experience sexual assault (Vázquez et al., 2012) and sex discrimination (sexism), and they may be expected to conform to gender roles that limit their educational opportunities. Moreover, female individuals with mental health challenges experience the added stigma of being a woman and also having psychological conditions (Meluch, 2025). In addition, recent research highlights the detrimental impacts of negative body image on female students’ mental health, aggravated by the societal norms portrayed through social media (Vajpayee, 2024). At the same time, female students are found to possess more advanced emotional intelligence (Brackett et al., 2004; Fida et al., 2018; Kant, 2019), and they utilize better coping skills when dealing with traumatic experiences (Banyard & Cantor, 2004). Additionally, compared to male students, female students with psychological disorders tend to rely more heavily on social support (Zhang et al., 2018), and they are more likely to engage in help-seeking behaviors to reduce mental stress (Johnson et al., 2023). Recent studies have found that female college students are more likely to seek professional mental health services than non-female students (Brand et al., 2019; Pedrelli et al., 2016; Seehuus et al., 2021). In summary, even though women often exhibit higher emotional intelligence and more resiliency, traits that are associated with seeking social and professional help for mental health challenges, evidence continues to show that female students are more likely to experience the negative outcomes related to psychological disorders. Therefore, taking a deeper dive into female students’ mental health challenges and help-seeking behavior when other important student-level factors (e.g., disability status, first generation college student status) are accounted for can be an important step in better understanding their mental health challenges and help-seeking behaviors.

### First Generation College Students

First generation college students (FGCS), or students whose parents did not attend college, often experience additional challenges when transitioning to postsecondary education. Compared to non FGCS, FGCS are more likely to have jobs to support themselves financially, and they often handle familial responsibilities for their extended family while in college (Martinez et al., 2012; Stebleton & Soria, 2013). They can also encounter a cultural mismatch between their home culture and the culture of the educational system, and this mismatch is associated with added psychological burdens (Phillips et al., 2020).

A recent review (Rockwell & Kimel, 2025) concluded that FGCS encounter heightened psychological challenges when academic and social experiences do not align with their cultural norms and interdependency, but when measured holistically, FGCS did not report significantly higher mental health challenges than non-FGCS. Another review (Smith & McLellan, 2023) found that while some studies reported heightened mental health issues among FGCS, other studies reported no significant differences or mixed findings. They also found contradictory results regarding FGCS’s help-seeking behaviors. This scoping review points out the need for more work to be done in better understanding the mental health challenges of FGCS and their help-seeking behaviors.

Regardless of such mixed findings related to their mental health, FGCS tend to display resiliency and optimism when dealing with difficult situations (Alvarado et al., 2017; Hammermeister et al., 2020), perhaps due in part to the strong social support they receive from their family and community (Azmitia et al., 2018). Such resiliency and social capital may buffer against their overall psychological well-being, in spite of their added layer of responsibilities and emotional burdens.

In sum, there is evidence that FGCS’s resiliency and social capital may function as protective factors during stressful times. Nevertheless, when FGCS do need support beyond what their social and familial support can offer, they may encounter barriers. More specifically, cultural stigma around accessing mental health services can play a role in whether or not FGCS access mental health services (Chang et al., 2020; Garriott et al., 2017).

All in all, considering such mixed findings on the level of mental health challenges among FGCS and their utilization of professional help, it would be worthwhile to take a look at the impact of FGCS status on college students’ mental health and their utilization of psychological services, including how these factors interact with other identities. While mental health and utilization of psychological services by college students with marginalized identities has been receiving attention in the recent literature base, the severity of mental health symptoms and students use of services, specifically among those with psychological disorders, is less explored. The present study seeks to begin to fill this gap in the literature.

Given that approximately one in five college students report psychological disorders (Auerbach et al., 2016), it would be important to examine student-level factors that are associated with the severity of symptoms among college students with psychological disorders. Additionally, considering the underutilization of mental health services among college students with psychological disorders, it would be equally worthwhile to investigate factors associated with accessing such services on campus. Therefore, the present study investigates the following research questions:

1. Among college students with self-reported psychological disorders, how do their marginalized identities (i.e, having autism, having ADHD, having both autism and ADHD, being female, having FGCS status) and their intersectionalities influence the severity of their mental health challenges?
2. Among college students with self-reported psychological disorders, how do their marginalized identities (i.e, having autism, having ADHD, having both autism and ADHD, being female, having FGCS status) and their intersectionalities influence their utilization of psychological services on campus?

We hypothesized that college students with traditionally marginalized identities who have psychological disorders would report more severe symptomology when compared to their peers with psychological disorders without these identities. We further hypothesized that college students with ADHD and female students may access mental health services on campus more than their counterparts, while autistic college students and FGCS would be less likely to utilize such services. We also hypothesized that those who are at the intersection of multiple marginalized identities would experience more severe psychological symptoms, and they would face additional barriers when accessing services.

## Methods

### Participants

The present study used data from the 2019 and the 2020 Diverse Learning Environment (DLE) national survey developed by the Cooperative Institutional Research Program at the Higher Education Research Institute (HERI) at the University of California, Los Angeles (HERI, 2022). The DLE is annually administered nationwide by 2-year and 4-year colleges to students who have obtained at least 24 credit hours at 2-year colleges and 2nd and 3rd year students at 4-year colleges. The DLE captures student perceptions of faculty, staff, and peers and also asks students to report on their school’s climate relating to diversity and student outcomes (HERI, 2022). As part of the survey, students are asked questions about their disabilities and mental health conditions.

All participating students were asked to select all that apply to the following question: “Do you have any of the following disabilities or medical conditions?” Response options included *psychological disorder (e.g., depression, anxiety, PTSD, etc.)*, among others. On the 2019 and 2020 surveys, 5,120 students from 32 schools reported a diagnosis of psychological disorder. Students were included in the present study if they self-reported a diagnosis of a psychological disorder (*N* = 5,120). Students in the sample completed the survey at one time during the survey years 2019 to 2020.

### Measures

#### Demographic Characteristics

Demographic characteristics of the sample included self-reported: (1) race/ethnicity, (2) gender, and (3) first-generation college student status.

### Student-Level factors

#### Race/ethncity

Students were asked, “Are you (select *all* that apply)” Response options included (1) American Indian non-Hispanic, (2) Asian non-Hispanic, (3) Black non-Hispanic, (4) Hawaiian non-Hispanic, (5) Hispanic - any race, (6) White non-Hispanic, (7) Two or more races non-Hispanic, and (8) Unknown. These options were recoded as a binary variable with “White non-Hispanic” as the reference group.

#### Gender

Students were asked, “What is your current gender identity?” Response options included (1) man/trans man, (2) woman/trans woman, (3) non-binary, (4) genderqueer/gender non-conforming, and (5) identity not listed above. These options were recoded as a binary variable with “woman/transwoman” as the reference group.

#### First-Generation College Student Status

Students were asked to indicate their first generation college student status as “yes” if their parents’ education level was less than “some college.” The response options included (1) yes or (2) no.

#### Autism and ADHD

Students were asked to select all that apply to the following question: “Do you have any of the following disabilities or medical conditions?” Response options included learning disability (e.g., dyslexia), attention-deficit/hyperactivity disorder (ADHD), autism spectrum disorder, physical or sensory disability (e.g., speech, sight, mobility, hearing), chronic illness (e.g., cancer, diabetes, autoimmune disorder), psychological disorder (e.g., depression, anxiety, PTSD), and “other”. To account for high probability of autism and ADHD co-occurring, The *Autism Only* variable was coded as 1 if the student reported having autism but no ADHD. Otherwise, it was coded as 0. Similarly, the *ADHD Only* variable was coded as 1 if the student reported having ADHD but no autism. Otherwise, it was coded as 0. *The Autism and ADHD* variable was coded as 1 if the student reported having autism and ADHD. Otherwise, it was coded as 0.

### Dependent Variables

#### Mental Health Challenges

To measure students’ mental health challenges, a latent variable was created from three items of students’ self-rated mental health challenges. Students were asked, *“Since entering college, how often have you: (1) felt overwhelmed by all you have to do, (2) felt depressed, and (3) felt anxious.”* Students rated the frequency of occurrence of each item. Response options included: (1) “Not at all”, (2) “Occasionally”, and (3) “Frequently”. Initially, there was another item in the model where students were asked to rate their emotional health on a scale of 1 to 5 (1—“Lowest 10%”; 2—“Below average”; 3—“Average”; 4—“Above average”; 5—“Highest 10%”). However, this item was removed from the final model due to low factor loading and high RMSEA. The unidimensional confirmatory model with the three remaining items was a saturated model with zero degrees of freedom. Cronbach’s alpha approached adequate internal consistency of the items (α = 0.693). Lastly, *Mental Health Challenges* factor scores based on this confirmatory model were calculated for each student in the sample.

Due to the categorical nature of these variables, diagonally weighted least squares (DWLS) were used to estimate the model parameters. Factor loading for the latent variable and reliability coefficient are summarized in Table 1.

**Table 1.** Factor Loadings and Reliability Coefficients for the *Mental Health* Latent Variable.

Mental health	
Items	Factor loadings
“Felt overwhelmed by all you have to do”	0.853
“Felt depressed”	0.752
“Felt anxious”	0.770
Cronbach’s α	.693

#### Psychological Services

To measure students’ utilization of psychological services, the survey asked, “*Since entering this college, how often have you utilized the following services?”* One of the items listed was “*student psychological services.”* Response options included: (1) “Not at all,” (2) “Occasionally,” and (3) “Frequently.”

Descriptive statistics and correlations of all study variables are illustrated in Tables 2 and 3.

**Table 2.** Descriptive Statistics for All Study Variables.

Variables	
Gender (woman/trans woman = 1) *n* (%)	3,697 (72.2%)
FGCS *n* (%)	1,006 (19.6%)
Autism only *n* (%)	65 (1.3%)
ADHD only *n* (%)	709 (13.8%)
Autism and ADHD *n* (%)	31 (0.6%)
Mental health challenges factor score *M* (*SD*)	0 (1.0)^a^
Psychological services *M* (*SD*)	1.46 (0.703)

a A factor score with the mean of zero and SD of 1.

**Table 3.** Correlations of Study Variables.

Variables	Woman	FGCS	Autism only	ADHD only	Autism and ADHD	Mental health challenges	Psychological services
Woman	1	−0.005	−.059**	−.063**	−.094**	.071**	0.006
FGCS		1	−.030*	−.053**	−0.014	−0.021	−.052**
Autism only			1	−.047**	−0.009	−0.004	−0.002
ADHD only				1	−.032*	.047*	0.024
Autism and ADHD					1	−0.014	0.003
Mental health challenges						1	.147**
Psychological services							1
*N*	5,081	5,030	5,007	4,992	5,002	3,039	5,081

* Correlation is significant at the 0.05 level (two-tailed).

** Correlation is significant at the 0.01 level (two-tailed).

### Analyses

#### Factors Associated with Heightened Mental Health Challenges

To test student-level factors that are associated with heightened mental health challenges among college students with psychological disorders, a multiple regression with interaction analysis was conducted with the entire sample (*N* = 5,120). The *Mental Health Challenges* factor score served as the outcome variable for the model, and the following variables entered the models as independent variables: Model (1) gender (woman as the reference group), first-generation college student status; Model (2) gender, first-generation college student status, autism only, ADHD only, autism and ADHD; Model (3) gender, first-generation college student status, autism only, ADHD only, autism and ADHD, autism*first generation interaction term, autism*ADHD interaction term, autism*gender (woman) interaction term, ADHD*gender(woman) interaction term.

#### Facilitators and Barriers to Utilization of Psychological Services

To test student-level factors that are associated with utilization of psychological services among college students with psychological disorders, a multiple regression with interaction analysis was conducted with the entire sample. The same set of independent variables entered the three models as described above. The outcome variable was the *Utilization of Psychological Services* variable.

All missing values were handled by listwise deletion. The Lavaan package (Rosseel, 2012) in R was used to estimate the structural equation model using the MLM (i.e., maximum likelihood) estimator to account for the violation of multivariate normality.

## Results

### Demographic Characteristics

Demographic characteristics of the sample are summarized in Table 4. The majority of students in the analytic sample were White (58.8%) and identified as a woman/trans woman (72.2%). Only a small portion of the sample (19.6%) were first-generation college students.

**Table 4.** Demographic Characteristics of the Sample.

Demographic characteristics	*N* (%)
	*N* = 5,120
Race	
White/non-Hispanic	3,010 (58.8)
Two or more races/non-Hispanic	765 (14.9)
Hispanic—any race	728 (14.2)
Asian/American Indian/Hawaiian	355 (7.0)
Black/non-Hispanic	231 (4.5)
Unknown	31 (0.6)
First generation college students	1,006 (19.6)
Gender identity	
Man/trans man	919 (17.9)
Woman/trans woman	3,697 (72.2)
Non-binary	115 (2.2)
Genderqueer/gender non-conforming	120 (2.3)
Identity not listed above	152 (3.0)
Annual family income	
under $30,000	1,486 (29.1)
$30,000–$50,000	775 (15.2)
$50,000–$100,000	1,420 (27.7)
$100,000–$200,000	959 (18.8)
$200,000 or more	353 (6.9)

### Mental Health Challenges

The *Mental Health Challenges* model is summarized in Table 5. When the students’ gender and FGCS status entered the model (Model 1), being a woman was associated with heightened mental health challenges (*β* = 0.066, *p* < .001). That is, students with psychological disorders who identified as a woman/trans woman were likely to experience greater challenges with their mental health. When the students’ gender, FGCS status, ASD, ADHD, and ASD & ADHD co-occurrence entered the model (Model 2), being a woman was associated with heightened mental health challenges, and so was having ADHD (*β* = 0.069, *p* < .001; *β* = 0.049, *p* = .009 respectively). When interaction terms (ASD*FGCS, ADHD*FGCS, ASD*Gender, ADHD*Gender) were added to the model (Model 3), being a woman continued to be associated with higher mental health challenges (*β* = 0.293, *p* < .014), but having ADHD was no longer a significant predictor. However, particularly for students with ADHD, being a FGCS was linked to higher mental health challenges (*β* = 0.128, *p* < .010).

**Table 5.** Multiple Regression Model Summary for *Mental Health.*

Outcome	Predictors	Standardized *β*	*S.E.*	*t*-ratio	*p*
	Model 1				
Mental health	Gender (woman = 1)	.066	0.040	3.596	**<**.001*
	First generation college student	−.020	0.0.047	−1.102	.271
	*R*^2^ = .005 *F* = 7.067 (*p* < .001)				
	Model 2				
	Gender (woman = 1)	.069	0.041	3.717	**<**.001*
	First generation college student	−.017	0.047	−0.908	.364
	ASD only	.003	0.165	0.152	.879
	ADHD only	.049	0.052	2.631	.009*
	ASD and ADHD	−.006	0.230	−0.352	.725
	*R*^2^ = .007 *F* = 4.258 (*p* < .001)				
	Model 3				
	Gender (woman = 1)	.293	0.263	2.452	.014*
	First generation college student	−.086	0.186	−1.172	.241
	ASD only	.029	0.243	1.060	.289
	ADHD only	−.009	0.091	−0.270	.787
	ASD and ADHD	−.011	0.287	−0.458	.647
	ASD × first gen	−.022	0.177	−0.288	.774
	ADHD × first gen	.128	0.080	2.578	.010*
	ASD × gender	−.172	0.235	−1.548	.122
	ADHD × gender	−.058	0.090	−1.027	.305
	*R*^2^ = .010 *F* = 3.453 (*p* < .001)				

* Relationship is statistically significant at the 0.05 level.

### Utilization of Mental Health Services

The *Utilization of Mental Health Services* model is summarized in Table 6. When the students’ gender and FGCS status entered the model (Model 1), being a FGCS was associated with less utilization of mental health services (*β* = −0.051, *p* < .001). That is, first-generation college students with psychological disorders were less likely to seek mental health services on campus. When the students’ gender, FGCS status, ASD, ADHD, and ASD & ADHD co-occurrence entered the model (Model 2), being a FGCS continued to be linked to less utilization of mental health services (*β* = −0.050, *p* < .001), and none of the other variables were. Even when interaction terms (ASD*FGCS, ADHD*FGCS, ASD*Gender, ADHD*Gender) were added to the model (Model 3), being a FGCS was the only predictor variable that was negatively associated with utilization of such services. No other variables had statistically significant relationship with the outcome variable in this model (Table 7).

**Table 6.** Multiple Regression Model Summary for *Utilization of Psychological Services.*

Outcome	Predictors	Standardized *β*	*S.E.*	*t*-ratio	*p*
	Model 1				
Utilization of mental health services	Gender (woman = 1)	.012	0.022	0.862	.388
	First generation college student	−.051	0.025	−3.601	**<**.001*
	*R*^2^ = .003 *F* = 6.869 (*p* = .001)				
	Model 2				
	Gender (woman = 1)	.014	0.023	0.975	.329
	First generation college student	−.050	0.025	−3.510	**<**.001*
	ASD only	.000	0.089	−0.011	.991
	ADHD only	.020	0.029	1.411	.158
	ASD & ADHD	.004	0.127	0.312	.755
	*R*^2^ = .003 *F* = 3.160 (*p* < .008)				
	Model 3				
	Gender (woman = 1)	−.036	0.136	−0.417	.677
	First generation college student	−.105	0.091	−2.016	.044*
	ASD only	−.023	0.123	−1.159	.246
	ADHD only	.021	0.050	0.828	.408
	ASD and ADHD	−.009	0.153	−0.537	.591
	ASD × first gen	.065	0.085	1.184	.237
	ADHD × first gen	−.005	0.043	−0.135	.892
	ASD × gender	.044	0.120	0.549	.825
	ADHD × gender	.010	0.050	0.221	.305
	*R*^2^ = .004 *F* = 2.088 (*p* < .027)				

* Relationship is statistically significant at the 0.05 level.

**Table 7.** Proportion of Non-White Students Among First Generation College Students (FGCS).

White vs. Non-White students	Non-FGCS (%)	FGCS (%)	Total
Non-White students	1,435 (35.3)	638 (63.4)	2,073
White students	2,627 (64.7)	368 (36.6)	2,995
Total	4,062	1,006	5,068
Pearson Chi-Square	263.236 (*p* < .001)		

## Discussion

Our study explored which student-level factors contributed to college students’ mental health challenges and their use of psychological services using a secondary dataset from a nationwide survey given to 2-year and 4-year colleges students. Using this dataset, first we looked for student-level factors that were associated with heightened mental health challenges for students with psychological disorders. Next, we looked for the factors that were associated with utilization of psychological services in the same group of students. Our major findings are discussed below.

### Female Students and/or First Generation College Students with ADHD Report More Mental Health Challenges

We found that, compared to male students with psychological disorders, female counterparts reported heightened mental health challenges, even after controlling for a number of other variables such as their disability and FGCS status. We also found that female students’ disability statuses (e.g., whether they have autism and/or ADHD) did not influence the level of mental health challenges they experience. These findings are consistent with research showing that female college students are more likely to report having psychological disorders, and, within the group that report having psychological disorders, female college students report worse mental health symptoms (Auerbach et al., 2019; Eisenberg et al., 2013; Grigsby et al., 2020; Sturm & Kasari, 2019). This is true even though women tend to display advanced coping skills (Banyard & Cantor, 2004), and they are able to rely on their social support when faced with stressful situations (Zhang et al., 2018). Although we captured their level of symptomology, we do not know the level of impairment, so social support may still be buffering their level of impairment. Nevertheless, female students are more vulnerable to severe forms of psychological disorders. One possible explanation could be that they are more likely to experience gender discrimination and sexual harassment (Latcheva, 2017; Wood et al., 2021), which may exacerbate psychological symptoms. Regardless of the cause, Institutions of higher education (IHE) must continue to work towards better supporting women with psychological disorders. For instance, considering female students’ reliance on their social support, creating peer support groups on campus for female students who are struggling with gender-related traumas could be one way to provide a more supportive learning environment for these students.

We also found that when the intersectionality of having ADHD and being a FGCS was not accounted for, it appeared that having ADHD itself was associated with increased mental health challenges among students with psychological disorders. However, when such intersectionality was accounted for, it was the dual identity of having ADHD and being a FGCS that contributed to heightened mental health challenges. This was indeed an interesting finding, as being a FGCS alone was not associated with more or less mental health challenges. Students with ADHD tend to struggle with self-directed learning and executive functioning skills, yet these skills are critical for success in college, so these challenges can cause students with ADHD to experience stress. FGCS with ADHD may feel even more stress because, like many FGCS, they worry more about paying for college (Costello et al., 2018), they are often unable to lean on their family for advice as they progress through college (Gibbons et al., 2019), and they may not feel like they belong at college (Costello et al., 2018). Further, they may feel some added stigma and stress if they are a member of a culture or community that holds negative views around ADHD diagnoses (Bailey et al., 2014; Feng et al., 2024; Schmitz & Velez, 2003). In sum, we can infer that, while FGCS may rely heavily on their familial and community support to cope with mental health challenges, if they feel that their disability status is not fully accepted by their own community, their psychological symptoms may worsen. Therefore, raising awareness and acceptance of neurodiversity on campus could help increase these students’ sense of belonging by creating a community of support.

### First Generation College Students Less Likely to Utilize Psychological Services

Even after controlling for a number of student-level factors and intersectionalities, our results showed that FGCS with psychological disorders were less likely to utilize psychological services on campus. This finding is concerning, given that approximately one-third of college students are FGCS (Cataldi et al., 2018), and FGCS face unique stressors (e.g., financial worries, academic stress, family obligations; House et al., 2020; Pratt et al., 2019; Stebleton & Soria, 2013) that may contribute to their overall well-being. Furthermore, many FGCS are not White or of European ancestry, and their families may have immigrated to the United States. Consequently, they are more likely to face additional challenges due to a cultural misalignment between their home culture, which is collectivistic, and the academic culture, which is strongly individualistic (Vasquez-Salgado et al., 2015). Additionally, FGCS have reported a lower sense of belonging on college campuses (Phillips et al., 2020), which can negatively impact well-being and academic achievement.

When it comes to seeking mental health services, ethnic minorities are more likely to report that there are barriers related to providers’ lack of cultural sensitivity that prevent them from seeking needed mental health treatment (Horwitz et al., 2020). Taken together, these findings suggest that psychological services tailored to FGCS that recognize and value their cultural capital, family and community support, and resiliency (Azmitia et al., 2018; Garriott et al., 2017; Hammermeister et al., 2020), while addressing their unique stressors (House et al., 2020; Stebleton & Soria, 2013) could be one important factor in helping FGCS with psychological disorders access and benefit from psychological services on campus.

### Recommendations for Institutions of Higher Education

Our findings suggest several areas where colleges and clinicians who provide psychological services at IHEs could better support students with mental health needs. First, IHEs could promote acceptance of neurodiverse students. Colleges that embrace neurodiversity by highlighting neurodiverse students and faculty and/or soliciting feedback from neurodiverse students when planning for campus initiatives (Leake & Stodden, 2014, Wilson & Dallman, 2024) signal to current and prospective students that their IHE is supportive and welcoming of neurodiverse students. These strategies are likely to reduce feelings of isolation and increase feelings of belonging on campus, which can improve outcomes for students with psychological disorders. Second, IHEs could focus on improving mental health services for all college students with psychological disorders. Given the growing need for mental health services for undergraduates (Oswalt et al., 2020), universities may need to come up with new and innovative ways to treat students. Telehealth/remote delivered therapy provided by mental health professionals may be one way to increase college students’ utilization of mental health services (Hersch et al., 2024). Colleges could also offer courses that teach evidence-based mental health skills. For example, students who took a three-credit “Wellness and Resilience Course” showed improved mental health after taking the course (Chugani et al., 2023). Finally, clinicians who provide psychological services at IHEs could provide more culturally informed services to support students who are FGCS and/or come from more collectivistic cultures (Azmitia et al., 2018; Garriott et al., 2017; Hammermeister et al., 2020). Taken together, there are several ways IHEs can improve outcomes for minoritized and/or marginalized students with mental health challenges.

## Limitations and Future Directions

This study has several limitations. First, the survey requires self-report, but men and individuals from certain cultures have reported stigma and privacy concerns related to reporting mental health issues (Horwitz et al., 2020). Consequently, participants from these groups may have under-reported their mental health challenges. However, the survey was confidential, which may have assuaged some of these concerns. Second, some of these data were collected during spring, 2020 when campuses began to shut down due to COVID-19. Studies have shown that mental health (i.e., anxiety, depression, and stress) were worse for college students during this time (Wang et al., 2021), so some of the reported mental health challenges may be higher than usual. Third, this study examined the role of gender in college students’ mental health challenges and utilization of therapeutic services on campus. However, we did not investigate such experiences in gender-diverse (e.g., non-binary, genderqueer, gender non-conforming) students. This was because the sample size of those who self-reported as gender-diverse was too small, and inferences made from such a small sample would raise questions regarding their validity. Existing studies do underscore the heightened psychological challenges that gender-diverse students experience (Lipson, Raifman, et al., 2019), and researchers and practitioners must continue to explore ways to adequately support this population. Further, the current study used women/transwomen as the reference group due to how the data was reported through the HERI surveys. Therefore, we were unable to parse out the responses between cisgender women and transgender women. However, a number of research studies have shown that transgender women encounter stigma-related stress, and such stress contributes to higher levels of mental health challenges (e.g., Yang et al., 2015). Consequently, findings from the current study must be interpreted with caution as they may not accurately reflect the experiences of transwomen with psychological disorders. In addition, the *Mental Health Challenges* variable was constructed from the following three indicator variables: *feeling overwhelmed, feeling depressed, and feeling anxious*, when psychological disorders may encompass a much wider range of symptoms. The survey instrument measured these three symptoms, and therefore we were unable to measure students’ mental health challenges beyond these three internalizing characteristics. However, according to the National Alliance on Mental Illness (NAMI, 2023), anxiety and depression are the most prevalent mental health conditions in the United States. Therefore, these three variables were used as a proxy for the sample’s mental health challenges.

Lastly, the current study did not investigate the role of race/ethnicity in college students’ mental health challenges and their utilization of on-campus services because we found that the majority of FGCS were non-White (Table 7), and such overlap may lead to multicollinearity issues.

Future research should examine factors that contribute to the heightened mental health challenges reported by college students with psychological disorders. These studies could benefit from a mixed-methods or qualitative approach to shed light on the nuanced causes of these challenges. Future studies could also examine interventions and treatments that help students with psychological disorders, especially treatments that focus on supporting FGCS, students with ADHD, and/or female students. Finally, addressing how institutions can improve mental health services, particularly for marginalized students with ADHD/autism, while removing any unintended barriers to psychological services, is an area for future research.
